# Cooperativity of imprinted genes inactivated by acquired chromosome 20q deletions

**DOI:** 10.1186/1868-7083-5-S1-S5

**Published:** 2013-08-19

**Authors:** Athar Aziz, E Joanna Baxter, Carol Edwards, Clara Yujing Cheong, Mitsuteru Ito, Anthony Bench, Rebecca Kelley, Yvonne Silber, Philip A Beer, Keefe Chng, Marilyn B Renfree, Kirsten McEwen, Dionne Gray, Jyoti Nangalia, Ghulam J Mufti, Eva Hellstrom-Lindberg, Jean-Jacques Kiladjian, Mary Frances McMullin, Peter J Campbell, Anne C Ferguson-Smith, Anthony R Green

**Affiliations:** 1 Cambridge Institute for Medical Research and Wellcome Trust/MRC Stem Cell Institute, UK; 2 Department of Hematology, University of Cambridge, Cambridge, UK; 3 Department of Hematology, Addenbrooke’s Hospital, Cambridge, UK; 4 Department of Physiology, Development, and Neuroscience, University of Cambridge, Cambridge, UK; 5 A*STAR Singapore Institute for Clinical Sciences, Brenner Centre for Molecular Medicine, Singapore; 6 Department of Zoology, University of Melbourne, Melbourne, Victoria, Australia; 7 Department of Hematological Medicine, King’s College London, London, UK; 8 Karolinska Institute, Department of Medicine, Division of Hematology, Karolinska University Hospital Huddinge, Stockholm, Sweden; 9 Hopital Saint-Louis, AP-HP, Centre d’Investigations Cliniques, Paris, France; 10 Université Paris 7 Denis Diderot, Paris, France; 11 Center for Cancer Research and Cell Biology, Queen’s University, Belfast, UK; 12 Wellcome Trust Sanger Institute, Hinxton, Cambridge, UK

## 

Large regions of recurrent genomic loss are common in cancers; however, with a few well-characterized exceptions, how they contribute to tumor pathogenesis remains largely obscure. Here we identified primate-restricted imprinting of a gene cluster on chromosome 20 in the region commonly deleted in chronic myeloid malignancies. We showed that a single heterozygous 20q deletion consistently resulted in the complete loss of expression of the imprinted genes L3MBTL1 and SGK2, indicative of a pathogenetic role for loss of the active paternally inherited locus. Concomitant loss of both L3MBTL1 and SGK2 dysregulated erythropoiesis and megakaryopoiesis, 2 lineages commonly affected in chronic myeloid malignancies, with distinct consequences in each lineage. We demonstrated that L3MBTL1 and SGK2 collaborated in the transcriptional regulation of MYC by influencing different aspects of chromatin structure. L3MBTL1 is known to regulate nucleosomal compaction, and we here showed that SGK2 inactivated BRG1, a key ATP-dependent helicase within the SWI/SNF complex that regulates nucleosomal positioning. These results demonstrate a link between an imprinted gene cluster and malignancy, reveal a new pathogenetic mechanism associated with acquired regions of genomic loss, and underline the complex molecular and cellular consequences of "simple" cancer-associated chromosome deletions.

